# Physical Workload Patterns in U-18 Basketball Using LPS and MEMS Data: A Principal Component Analysis by Quarter and Playing Position

**DOI:** 10.3390/s25196253

**Published:** 2025-10-09

**Authors:** Sergio J. Ibáñez, Markel Rico-González, Carlos D. Gómez-Carmona, José Pino-Ortega

**Affiliations:** 1 Research Group in Optimization of Training and Sports Performance (GOERD), Department of Didactics of Music, Plastic and Body Expression, Faculty of Sport Science, University of Extremadura, 10003 Caceres, Spain; sibanez@unex.es; 2 Department of Music, Plastic and Body Expression, University of the Basque Country, 48940 Leioa, Spain; markel.rico@ehu.eus; 3 Research Group in Training, Physical Activity and Sports Performance (ENFYRED), Faculty of Human and Social Sciences, University of Zaragoza, 44003 Teruel, Spain; 4 BioVetMed & SportSci Research Group, Department of Physical Activity and Sport, University of Murcia, 30100 Murcia, Spain; josepinoortega@um.es

**Keywords:** workload monitoring, team sports, performance analysis, wearable sensors, ultra-wideband positioning, inertial measurement units, multivariate statistics

## Abstract

**Highlights:**

**What are the main findings?**

- High-intensity variables (e.g., accelerations, explosive distance) were identified in early quarters and declined progressively, with 5–8 components explaining 61–73% of the variance.
- Position-specific profiles emerged: guards exhibited frequent accelerations and direction changes, forwards engaged in mixed-intensity efforts, and centers experienced a high number of impacts and jumps.

**What are the implications of the main findings?**

- LPS and MEMS data, combined with PCA, enable basketball teams to identify the most important workload parameters and specific profiles based on contextual factors.
- Findings support individualized training prescriptions and injury prevention by understanding the dynamic nature of basketball demands during competition.

**Abstract:**

Basketball is a high-intensity, intermittent sport in which physical demands fluctuate depending on different contextual variables. Most studies addressed these demands in isolation without integrative approaches. Therefore, the present study aimed to identify key variables explaining players’ physical workload across game quarters and playing positions through principal component analysis (PCA). Ninety-four elite U18 male basketball players were registered during the EuroLeague Basketball ANGT Finals using WIMU PRO™ multi-sensor wearable devices that integrate local positioning systems (LPS) and microelectromechanical systems (MEMS). From over 250 recorded variables, 31 were selected and analyzed by PCA for dimensionality reduction, analyzing the effects of game quarter and playing position. Five to eight principal components explained 61–73% of the variance per game quarter, while between four and seven components explained 64–69% per playing position. High-intensity variables showed strong component loadings in early quarters, with explosive distance (loading = 0.898 in total game, 0.645 in Q1) progressively declining to complete absence in Q4. Position-based analysis revealed specific workload profiles: guards required seven components to explain 69.25% of the variance, with complex movement patterns, forwards showed the highest explosive distance loading (0.810) among all positions, and centers demonstrated concentrated power demands, with PC1 explaining 34.12% of the variance, dominated by acceleration distance (loading = 0.887). These findings support situational and individualized training approaches, allowing coaches to design individual training programs, adjust rotation strategies during games, and replicate demanding scenarios in training while minimizing injury risk.

## 1. Introduction

Basketball is a high-intensity intermittent sport where repeated explosive actions are combined with short recovery periods. These demands are modified according to factors such as player position, game period, competitive level, and individual characteristics of the basketball players [1,2,3]. The efficient performance of specific movements like accelerations, changes in direction, jumps, and defensive or offensive displacements is a determinant for winning [4]. In this context, analysis of the most demanding scenarios (MDS) allows for the identification of game periods with high-intensity demands [5]. This has enabled the identification of variations in these scenarios throughout the game, with notable differences between quarters and positions [6,7,8]. Understanding these dynamics is essential for designing training that replicates the demands during competition.

In the last decade, a rapid evolution of external load monitoring in basketball has occurred due to the incorporation of wearable technologies such as local positioning systems (LPS) and microelectromechanical systems (MEMS, generally including accelerometers, gyroscopes, and magnetometers) [9,10,11]. These technologies have allowed us to overcome the limitations of average-based analysis, which precisely identifies the intensity peaks in specific game periods [12,13]. Physical demands present fluctuating dynamics influenced by fatigue accumulation and tactical decisions, with various studies showing a progressive decrease in external load throughout the encounter [14,15,16]. This trend reflects the physiological dynamics of this fatigue accumulation, which include progressive increases in heart rate reaching an 85–95% maximum by the fourth quarter, blood lactate concentrations peaking at 4–8 mmol·L^−1^ during middle quarters, and declining neuromuscular function evidenced by reduced jump performance [17,18,19].

Also, MDS varies according to game quarter and player position, with higher demands reported for guards at the beginning of the game and for centers in the final quarters [20,21]. Generally, competition demands decrease throughout the game for players [22,23] and referees [24,25], motivated by basketball game dynamics and increased interruptions. However, different methodological challenges persist that limit study comparisons, such as the standardizing of intensity thresholds [26,27] and classification of playing positions [28]. Recent research has explored external and internal load relationships by game quarter and position. In this sense, Yang [29] observed significant differences between guards and centers, highlighting the need for integrated load monitoring. Additionally, contextual factors, such as score, team strategy, or game period, influence these demands, promoting individualized analytical approaches [13].

In this context, player positions also have a direct effect on physical demands in basketball. Guards and shooting guards presented with higher values in accelerations, changes in direction, and high-intensity displacements. On the other hand, centers and power forwards suffered more impacts and jumps due vertical–horizontal explosive actions [14,30,31]. This specialization generates specific load profiles that must be considered when designing training and recovery programs. For example, guards achieve acceleration/deceleration ratios superior to 3 m·s^−2^, reflecting high neuromuscular stress linked to pace control and constant changes in direction [15,32,33]. Studies in young players have shown that physical demands vary significantly by playing position, even within the same age category. For example, interior players tend to present with higher loads derived from physical contact, while guards must sustain more prolonged intermittent efforts [34,35,36]. The use of this information by the team allows for more precise training management and more effective preventive planning.

Basketball is characterized by high-intensity movements in a reduced area, making a multivariable analytical approach necessary to understand its physical demands. Principal Component Analysis (PCA) has emerged as an effective tool to reduce the complexity of the data registered by Electronic Performance and Tracking Systems (EPTS). This technique allows for the identification of a limited set of components that explain most of the variance in external load [30,37,38]. PCA previously demonstrated effectiveness in reducing large quantities of data (e.g., from over 200 variables to fewer than 10 components) while preserving maximum variance. Also, PCA identifies latent patterns without making prior assumptions about variable relationships, and allows for an exploratory identification of natural groupings in physical demands [37]. Different variables, such as accelerations, jumps, the speed of displacements, and relative player load, have been identified as key indicators of external workload during competition [23,32,39,40].

The use of PCA has been validated in multiple team sports, including soccer, rugby, and futsal. It has synthesized eight to ten metrics into three or four principal components that explain more than 70% of external workload dynamics [15,35,36]. Its application in basketball has shown utility for identifying load profiles according to playing position, game quarter, and competitive level [41,42]. Despite advances in understanding basketball’s physical demands, there is a limited integration of multivariable methodologies that allow for the simultaneous analysis of external load fluctuations according to playing time and specific player position. Most studies have focused on isolated descriptive or comparative analyses, without considering the dynamic complexity involved in players’ physical behavior during a complete game. In this context, PCA presents itself as a valid strategy to reduce data dimensionality and detect relevant patterns that explain physical load from a situational and positional approach.

Therefore, the main objective of this study was to identify the main variables that explain basketball players’ physical load according to game moment and field position using principal component analysis. We also established the following specific aims: (i) analyze external load principal components according to game quarters; (ii) examine differences in load profiles according to playing position (guards, forwards, and centers); (iii) determine common and divergent patterns between both approaches (temporal and positional) that allow for training strategies to be designed that are more adapted to real competition demands. We hypothesized that (1) high-intensity variables will dominate early quarters, with a progressive decline; (2) playing positions will show distinct component structures reflecting role-specific demands; and (3) component complexity will vary by quarter and position.

## 2. Materials and Methods

### 2.1. Study Design

A descriptive–comparative design was conducted during the finals of the Basketball Euroleague ANGT tournament [43]. WIMU PRO^TM^ devices using a local positioning system (LPS) and microelectromechanical systems (MEMS) were utilized to monitor physical load variables. Through an exploratory factor analysis, the most relevant variables associated with players’ workload dynamics were identified. Finally, these workload variables were compared according to game quarter and playing position.

### 2.2. Participants

A total of 94 male elite basketball players under 18 years of age participated in this study (mean age: 17.6 ± 0.8 years; mean height: 1.91 ± 0.08 m; mean body mass: 82.5 ± 8.8 kg; mean BMI: 22.7 ± 1.8 kg/m^2^). Players belonged to eight teams that participated in the Euroleague Basketball ANGT finals tournament. The distribution by playing positions consisted of guards (*n* = 34), forwards (*n* = 47), and centers (*n* = 13).

Before data collection, all participants received comprehensive information regarding the research objectives, methodological procedures, and their rights. As all players were over 16 years old and they signed a written informed consent before the tournament started. The research protocol received ethical approval from the Bioethics Committee of the University of Extremadura (approval reference: 67/2017) and adhered to the ethical standards established by the Declaration of Helsinki for human research [44]. Additionally, formal authorization was secured from both teams’ staff and tournament organizers for participation in this research.

### 2.3. Instruments

Player positioning was recorded using a tracking system that combined a local positioning system (LPS) based on ultra-wideband (UWB) technology with a WIMU PRO™ microelectromechanical system (RealTrack Systems, Almería, Spain). The UWB technology, operating on a bandwidth exceeding 0.5 GHz, has demonstrated high precision even in multipath contexts, such as when multiple devices operate simultaneously [45]. The 70 g device (81 × 45 × 16 mm^3^) incorporates a microprocessor, 8 GB flash memory, and a high-speed USB interface for data storage and transfer.

### 2.4. Procedures

The methodology was designed following the guidelines proposed by Rico-González et al. [46] to ensure a rigorous description of technological implementation. Regarding the UWB-based system for LPS, 21 of the 23 established criteria were fulfilled. For the MEMS sensors, 17 of the 20 methodological criteria were documented. The remaining criteria could not be reported due to proprietary manufacturer information not being publicly available (e.g., internal sensor fusion algorithms, detailed environmental monitoring protocols during data collection).

#### 2.4.1. Ultra-Wide Band System

Data collection was conducted in a controlled environment, free from metallic structures that could interfere with signal transmission. The system consisted of an antenna network that transmitted and received radiofrequency signals, enabling device position calculation through the time difference of arrival (TDOA) technique. A five-minute stabilization protocol was observed to ensure the functioning of the system. Time synchronization was managed by the master antenna, which unified the temporal signal for all devices. Once activated at the system center, automatic one-minute synchronization was performed between antennas and devices. Speed was estimated using differential Doppler technology, which calculates velocity from frequency shifts in radiofrequency signals caused by player movement (Doppler effect), and acceleration was derived from these measurements. To prevent erroneous recordings, the manufacturer established minimum duration and velocity thresholds for measured movements, thus eliminating potential outlier values. Data were recorded at a sampling frequency of 18 Hz, which represents the standard UWB sampling frequency of the WIMU PRO™ system when configured with six antennae. System validity and reliability were previously confirmed in indoor environments [45]. This frequency exceeds the minimum 15 Hz sampling rate required for sports positioning applications [47], representing an optimal value to prevent precision losses associated with lower frequencies, while environmental conditions (temperature, humidity, and ventilation) were maintained stable to guarantee positioning reliability.

#### 2.4.2. Microelectromechanical System

The WIMU PRO™ device (RealTrack Systems, Almería, Spain) incorporates four triaxial accelerometers (±16 g, ±16 g, ±32 g, and ±400 g full-scale ranges), three triaxial gyroscopes (±8000°/s full-scale range), and a triaxial magnetometer, utilizing micro-electromechanical systems (MEMS) technology with adjustable sampling frequency from 10 to 1000 Hz. Each device features integrated signal filtering algorithms optimized for different sampling frequencies to ensure data quality. The validity of this MEMS-based system was previously established by Gómez-Carmona et al. [48] and Granero-Gil et al. [49]. The device was positioned on the upper back between vertebrae T2 and T4, secured within a fitted pocket of a custom elastic vest to minimize movement artifacts and ensure consistent data collection. Each player wore the same individually assigned vest throughout all games to maintain positioning consistency and device stability.

### 2.5. Variables

From the more than 250 variables recorded by the inertial devices, 31 relative metrics were selected after applying principal component analysis (PCA). These variables were chosen for their capacity to significantly represent physical load during gameplay. To facilitate comparison across different quarters and throughout the complete game, those related to effort, time, and maximum values achieved were prioritized.

Table 1 presents the variables included in the study and their abbreviations.

### 2.6. Data Processing and Statistical Analysis

Data were recorded in real-time and stored in the device’s internal memory during gameplay sessions. Upon completion of each session, data were downloaded from the WIMU PRO™ devices and imported into SPRO software (version 956, RealTrack Systems, Almería, Spain) for comprehensive data analysis and variable extraction. Following processing in SPRO, data were exported in Excel format and subsequently imported into SPSS software (IBM, SPSS Statistics, v.22.0, Chicago, IL, USA) for all conducted analyses.

The criterion to include players in the statistical analysis was participation in >60% of total playing time per quarter (including within-quarter breaks, e.g., free-throws, fouls, ball out, and others), except time-outs and between-quarter breaks. This threshold ensures statistical reliability by focusing on players with substantial game involvement, preventing brief performances from distorting results and ensuring that analyzed metrics validly reflect the actual competitive demands, as previously employed in basketball external load research [23,35,50,51]. The final sample by playing positions consisted of guards (*n* = 374), forwards (*n* = 466), and centers (*n* = 154), and the final sample by quarters consisted of Q1 (*n* = 263), Q2 (*n* = 269), Q3 (*n* = 249), and Q4 (*n* = 253). Notably, the positional distribution was not predetermined by the researchers but reflects the roster configuration of participating teams, where guards and forwards naturally outnumber centers according to league-specific technical–tactical performance requirements, as previous research found [52].

Then, Principal Component Analysis (PCA) was conducted following the methodological recommendations proposed by Rojas-Valverde et al. [41]. Data normality was assessed using the Shapiro–Wilk test, and variables showing parametric distributions (*p* > 0.05) were analyzed using Pearson correlation coefficients in the correlation matrix to identify redundancies. Those with null variance were excluded, and only variables showing correlations with r < 0.7 were retained [53]. Subsequently, the selected variables (between 21 and 22) were centered and scaled using Z-scores.

Model adequacy was verified through the Kaiser–Meyer–Olkin index (KMO = 0.64–0.78) and Bartlett’s sphericity test (*p* < 0.05), indicators that confirmed the suitability of PCA. These KMO values, while acceptable for PCA, reflect moderate correlations between variables, providing appropriate context for component interpretation. Component retention was determined using the Kaiser criterion (eigenvalues > 1), supplemented by scree plot examination and parallel analysis, to validate that retained components explained significantly more variance than random data matrices of equivalent dimensions, thereby reducing the risk of over- or underestimation [37,41]. Components with eigenvalues greater than 1 were extracted, and orthogonal Varimax rotation was applied to improve factor interpretability. Factor loadings > 0.60 were considered significant, following established guidelines for sports science PCA applications [41]. For cross-loadings (>0.60 on multiple components), variables were assigned to the component with the highest loading when the difference exceeded 0.10; otherwise, variables were excluded [54].

## 3. Results

### 3.1. PCA by Total Game and Quarters

Table 2 presents the principal component analysis effects of the total game and per quarter on physical load variables. High-intensity variables demonstrated reduced contribution to variance explanation in later quarters and were substituted by landing/takeoff and low-intensity movement patterns. During total game analysis, PC1 explained 21.99% of the variance, which was dominated by explosive distance (loading = 0.898), moderate-intensity decelerations (Dec Abs −3 to −2 m/s^2^, loading = 0.775), and impacts (3–5 G/min, loading = 0.775). This defined a definite high-intensity movement profile for the overall game structure.

During quarter analysis, Q1 was characterized by high-intensity activity with PC1 (21.38% variance), defined by explosive distance (loading = 0.645), distance covered during acceleration (loading = 0.719), moderate-intensity accelerations (Acc Abs 3–4 m/s^2^, loading = 0.838), and moderate decelerations (Dec Abs −4 to −3 m/s^2^, loading = 0.741). The landing variables were seen in PC2, with landing (3–5)/min (loading = 0.778) and landing (5–8)/min (loading = 0.755). Acceleration frequency was observed in PC3 (Acc/min, loading = 0.849). Q2 revealed the first significant structural change, with PC1 (18.51% variance) now dominated by landing and takeoff variables: takeoff (3–5)/min (loading = 0.835), landing (3–5)/min (loading = 0.772), and landing (5–8)/min (loading = 0.702). High-intensity variables became secondary components, with acceleration frequency (Acc/min, loading = 0.922) and low-intensity velocity (Vel Abs 0–6 m/min, loading = 0.892) characterizing PC2. Explosive distance shifted to PC3 (loading = 0.722) along with high-intensity velocity (Vel Abs 18–21 m/min, loading = 0.811).

In Q3, the direction changed toward low-intensity movement patterns. PC1 (31.25% variance) was defined by acceleration frequency (Acc/min, loading = 0.892), very-low-intensity accelerations (Acc Abs 0–1 m/s^2^, loading = 0.946), very-low-intensity decelerations (Dec Abs −1 to 0 m/s^2^, loading = 0.957), and low-intensity velocity (Vel Abs 0–6 m/min, loading = 0.767). Meanwhile, explosive distance fell to PC3 (loading = 0.834) alongside high-intensity velocity and distance during acceleration. Q4 exhibited the most extreme change in load structure, with PC1 (24.21% variance) entirely dominated by landing and takeoff variables: takeoff (3–5)/min (loading = 0.842), landing (3–5)/min (loading = 0.798), takeoff (5–8)/min (loading = 0.735), and landing (5–8)/min (loading = 0.732). Notably, explosive distance was absent from the component structure, while moderate-intensity accelerations (Acc Abs 3–4 m/s^2^, 4–5 m/s^2^, and 5–6 m/s^2^) were relegated to PC2.

### 3.2. PCA by Playing Position

Principal component analysis revealed distinct load patterns for centers, forwards, and guards, with specific movement profiles based on their tactical positions (Table 3). Centers displayed concentrated load patterns, with PC1 accounting for 34.12% of the variance. This was characterized by high-intensity velocity (Vel Abs 18–21 m/min; loading = 0.772), acceleration distance (loading = 0.887), and moderate accelerations (Acc Abs 3–4 m/s^2^/min; loading = 0.892), suggesting forceful movements across small spaces. PC2 included acceleration frequency (loading = 0.867) and moderate decelerations (loading = 0.894), while PC3 was dominated by centripetal force variables (loading = 0.747–0.762). The cumulative variance for the four components was 64.61%.

Forwards had the most explosive profile, with PC1 (30.36% variance) characterized by explosive distance (loading = 0.810)—the highest of all positions. This component also included high-intensity velocity (loading = 0.760), acceleration distance (loading = 0.736), and moderate accelerations (loading = 0.733). PC2 consisted of landing and takeoff variables (loading = 0.741–0.831), reflecting demands for aerial movements, and PC3 showed moderate deceleration patterns (loading = 0.739). Five-component cumulative variance was 65.12%.

Guards had the most complex load pattern, with PC1 explaining 24.75% variance but being dominated by moderate accelerations (loading = 0.864). PC2 included impact variables (loading = 0.761) and centripetal force measures (loading = 0.774–0.781), and PC3 included landing and takeoff patterns (loading = 0.701–0.831). Guards required seven components to achieve 69.25% cumulative variance, reflecting the need for extended, varied movement patterns.

### 3.3. Practical Interpretation of PCA

The PCA can characterize distinct physical demand profiles of basketball performance. In the total game analysis, PC1 represents an “explosive profile” dominated by explosive distance with moderate-intensity decelerations and impacts. PC2 constitutes a “speed changes frequency profile”, emphasizing the rate of accelerations and decelerations. PC3 forms a “vertical jump profile” characterized by landing and takeoff variables at different intensities, while PC4 represents an “interior game profile” formed by high-intensity impacts and maximum centripetal force to change in direction into the paint.

Regarding quarter-specific analysis, a systematic evolution of these demand profiles throughout the game was revealed. First quarter represents a “power profile” characterized by explosive and acceleration distance, as well as moderate-intensity accelerations and decelerations. This profile reflects counterattacks, turnovers, and aggressive physical actions when players are without fatigue. On the other hand, the second and fourth quarter shift to a “jump profile”, where landing and takeoff variables become dominant, indicating periods with more open throws, free throw situations, and vertical play as tactical approaches emphasize shooting rather than explosive ground movements. Finally, the third quarter tries to achieve the same power-based tendency as Q1, but fatigue prevents players from reaching the same explosive demands. Therefore, teams need to use more systematic approaches and structured offensive systems to achieve scoring opportunities rather than relying on individual explosive actions.

The analysis of playing positions revealed distinct profiles based on role demands. Centers’ PC1 represents a “power profile” characterized by high-intensity velocity during basket-to-basket transitions and acceleration distance in limited space, based on their interior-focused role. Forwards’ PC1 constitutes an “explosive profile” showing the highest explosive distance values among positions, combined with high-intensity velocity patterns that reflect their versatile court coverage and the search for free spaces to execute open shots. Finally, Guards’ PC1 and PC2 form a “complex agility profile” dominated by moderate accelerations, low impacts and high centripetal force to manage the direction of the game in the attack phase and to be aggressive in the defense phase. Due to the complexity of their role, seven components were required to capture their varied movement demands, compared to four to five components for other positions.

The transformation from explosive power-based demands in Q1 to aerial activity patterns in Q2 and Q4, with Q3 showing similar power intentions limited by accumulated fatigue, demonstrates how game flow, tactical decisions, and physiological state influence the fundamental nature of physical demands throughout basketball competition.

## 4. Discussion

The analysis of physical load in basketball revealed the complexity of specific physical demands derived from playing position and game period. The results from this study show that the intensity demands decrease throughout game quarters. In the final quarter, the explosive distance completely disappeared from Q4’s component structure after explaining 21.99% of the variance in the analysis of total game demands. Similarly, it is important to consider the effect of playing positions, such as guards, forwards, and centers, which require differentiated load profiles.

Similar findings have been reported in previous studies, with decreases in accelerations and decelerations as the game progresses [22,55] in both kinematic and neuromuscular terms due to the increasing interruptions. The systematic reorganization from a high-intensity dominant profile (Q1: PC1 = 21.38%) to a landing/takeoff dominant profile (Q4: PC1 = 24.21%) reveals a fundamental change in physical demands. This supports the cumulative impact of fatigue. Yang [29] also documented that while guards had a greater load per minute in the first quarter, the centers increased their physical involvement (more jumps and high-intensity accelerations) in the final quarters. They also showed some relative consistency in load in the intermediate quarters, which may be due to tactical considerations and players’ energy-efficiency.

Regarding playing positions, the results highlight distinct demands. Centers experienced a higher level of impact and centripetal force. This could be produced due to the number of times they were involved in physical contact within the paint area [35,39,56]. Forwards combined the use of medium- and high-intensity efforts in their role, establishing offensive and defensive transitions [2,23,36]. Guards exhibited a playing profile that was built on a high incidence of accelerations and directional changes. This indicates the need for specific work on agility and recovery capacity [4,31,32]. The high-intensity variables reflected a descending pattern that supports previous evidence of a significant and moderate physical decrement in load encountered during the game. This aspect may be attributable to cumulative fatigue [6,22,57], and is also specific to each role [34,58].

The principal component analysis (PCA) method helped determine load profiles for each quarter. This applies to the team sports context as, through PCA, it reduced the complexity of the amount of data and visually highlighted the most useful variables for performance [37,41]. Our study found an explained variance from 61 to 73% across quarters with four to eight principal components. These results align with previous systematic reviews where basketball studies generally achieved 62–89% of explained variance and required 3–7 principal components [37]. Similar approaches have been successfully implemented in soccer and rugby [38,42,59]. The PCA allowed us to highlight discriminative components for each quarter and position, substantiating the need to consider load profiling and monitoring from situational and player perspectives. However, the remaining 27–39% unexplained variance suggests that unmeasured factors such as tactical decisions, individual player characteristics, psychological factors, and other contextual variables likely contribute to the complexity of basketball performance. Consistent with prior studies, these findings demonstrated variations in external load between different high-intensity activities. Pernigoni et al. [60] observed that sprints and specific movements present greater accumulated load than jumps. In the same direction, Svilar et al. [36] and Garcia et al. [14] identified positional differences and varying demands across game scenarios. These outcomes coincided with our PCA, which showed components associated with speed, acceleration, and explosive movement that explained the variance in the first quarters, supporting previous research on high-intensity actions in basketball [22,34,40].

The application of technology such as UWB positioning and microelectromechanical sensors (MEMS) enables the exact capture of physical demands in real game situations [9,10,11]. The previous studies highlighted in the introduction used both technologies, establishing their efficacy in competitive environments, which adds to the robustness of the data obtained in this research [45,61,62]. Their integration with advanced statistical methods, such as PCA, provides a better analytical framework for decision-making in the training domain [30,37,38].

Furthermore, the PCA approach should acknowledge that basketball-specific tactical systems and contextual factors significantly influence physical load patterns, which may affect variable selection and component structure interpretation. Recent research has demonstrated that defensive strategies create different external workload demands, with zone defense being less physically demanding than man-to-man, while full-court press systems generate higher loads without affecting total distance covered [63,64,65]. Additionally, contextual factors such as game outcome and score differentials substantially modify both external and internal workload demands, with players exhibiting greater jumps, high-intensity accelerations and decelerations during losses, and unbalanced games presenting higher effective on-court time with different internal load responses through heart rate and perceived exertion [22,23,29,34,66]. Basketball offensive systems also impact load distribution, with pick-and-roll tactical adjustments producing different defensive responses that modify movement patterns [63,67]. This sensitivity of PCA to both tactical and contextual variables suggests that future basketball load monitoring should incorporate defensive schemes (man-to-man, zone, press), offensive systems (pick-and-roll frequency), and contextual factors (score differential, game outcome) to provide a comprehensive understanding of the multifactorial influences on load variance in competitive basketball.

Given the impact of fatigue and load variability during the game, these findings suggest the potential value of considering position-specific approaches to rotation, recovery, and training strategies. As different authors mentioned previously [42,68,69], continuous load monitoring allows for performance optimization and injury risk reduction. In this regard, adapting training to the demands detected by positions and periods could improve functional recovery and maintain performance throughout the competition [56,70].

This research has notable limitations that need to be acknowledged for the results to be accurately interpreted. The sample was limited to elite U-18 male basketball players, significantly restricting generalizability to female players, younger age groups, or recreational-level athletes who may exhibit different physiological responses, movement patterns, and physical demands. Future research should expand to include female basketball players and diverse age groups to establish comprehensive load profiles across different populations. The uneven distribution of players across positions (guards *n* = 34, forwards *n* = 47, centers *n* = 13) may introduce bias in position-specific analyses, particularly affecting the statistical power and generalizability of findings for centers. Future studies should aim for more balanced positional samples or employ stratified sampling approaches to enhance the reliability of position-specific load profile analyses.

The analysis was limited to games within an international tournament (Euroleague Basketball ANGT), which introduces contextual variability based on competition type, playing style, or tactical strategies. Future research should replicate this study across diverse competitive contexts, including professional leagues, formative levels, and national tournaments, to evaluate the consistency of identified load patterns. While principal component analysis (PCA) provides a useful way to represent complex data, the analytical process involves technical decisions that could condition subsequent interpretation. However, the remaining 27–39% unexplained variance suggests that unmeasured factors such as tactical decisions, individual player characteristics, psychological factors, and other contextual variables likely contribute to the complexity of basketball performance.

This proportion of unexplained variance is inherent to the complexity of PCA in team sports contexts and remains consistent with the range reported in previous basketball studies (62–89% explained variance) [37]. It underlines the multifactorial nature of basketball performance, where tactical systems (e.g., man-to-man vs. zone defense, offensive schemes), individual characteristics (e.g., anthropometry, fitness status), psychological variables (e.g., motivation, decision-making), and contextual influences (e.g., score differential, game outcome, rotation patterns) all interact to shape players’ workload. Acknowledging these unmeasured factors highlights the limitations of PCA while also pointing to future research directions, such as combining PCA with complementary approaches (e.g., cluster analysis, machine learning), to capture more fully the dynamic and complex determinants of performance in basketball.

Strategic decisions by coaching staff during games, such as rotations or tactical adjustments, which can significantly influence individual physical load, were not considered. Future research should integrate coaching decision-making patterns and their impact on player workload to provide a more comprehensive understanding of load management. Finally, combining PCA with complementary analytical methods such as cluster analysis and machine learning algorithms could enhance the sensitivity of multivariate analysis and detect complex interactions that traditional approaches might miss [71,72,73,74]. For instance, integrating PCA with k-means clustering has been successfully applied in basketball to categorize players into distinct performance profiles beyond conventional positional classifications, while neural networks utilizing PCA-reduced dimensionality have improved team roster construction and led to game outcome predictions with greater accuracy than single-method approaches [75,76].

## 5. Conclusions

This study demonstrates the need to adapt training strategies and load management based on the specific demands of each playing position and game quarter. The analyses show that high-intensity demands (e.g., maximum velocity and accelerations) are more pronounced in the first quarters and progressively decrease throughout the game, suggesting a cumulative effect of fatigue. External load profiles vary significantly according to player position: centers presented greater impacts and centripetal forces due to their location near the basket, forwards present a mixed profile combining explosive efforts and medium-intensity movements, while guards assume high demands for agility and changes in direction. The use of PCA enabled the identification of the most representative variables of external workload according to game quarter and playing position, offering an effective strategy for interpreting large volumes of data and providing practical value for designing evidence-based training to maximize performance and reduce injury risk.

The results suggest potential implications for training. Regarding playing positions, the distinct load profiles observed indicate that guards may benefit from an emphasis on agility and directional changes in training, centers may require focus on contact management and impact tolerance, and forwards may need training addressing their hybrid role demands. Regarding game quarters, the decrease in high-intensity demands in final quarters suggests distributing training load considering physical variability throughout the game, including active recovery strategies and adapting volume and intensity to achieve more stable performance. During official games, the understanding of load variations by quarters and playing positions may inform coaches’ decisions regarding rotations and tactical approaches, preserving physical performance and reducing injury risk. Additionally, PCA may potentially be integrated into load monitoring systems for data-based decision-making and personalized microcycle design. This approach could also allow for the replication of high-demand competitive contexts during training sessions to increase potentially increase stimulus specificity and transfer to competitive performance.

## Figures and Tables

**Table 1:** Variables and abbreviations.

Abbreviation	Unit	Variable
Expl Dist	m	Distance covered at explosive intensity
Vel Abs (0–6) (m/min)	m/min	Relative distance covered from 0 to 6 m/min
Vel Abs (18–21) (m/min)	m/min	Relative distance covered from 18 to 21
Vel Abs (21–24) (m/min)	m/min	Relative distance covered from 21 to 24 km/h
Vel Max	km/h	Maximum velocity achieved by a player
Acc/min	n/min	Number of accelerations per minute
Dist. Acc	m	Total distance covered accelerating
MAX Acc (m/s^2^)	m/s^2^	Maximum acceleration
Acc Abs (0–1)/min	n/min	Absolute accelerations lower than 1 m/s^2^ per minute
Acc Abs (1–2)/min	n/min	Absolute accelerations from 1 to 2 m/s^2^ per minute
Acc Abs (3–4)/min	n/min	Absolute accelerations from 3 to 4 m/s^2^ per minute
Acc Abs (4–5)/min	n/min	Absolute accelerations from 4 to 5 m/s^2^ per minute
Acc Abs (5–6)/min	n/min	Absolute accelerations from 5 to 6 m/s^2^ per minute
Acc Abs (6–10)/min	n/min	Absolute accelerations from 6 to 10 m/s^2^ per minute
Dec Abs (−1, 0)/min	n/min	Absolute decelerations lower than 1 m/s^2^ per minute
Dec Abs (−2, −1)/min	n/min	Absolute decelerations from 1 to 2 m/s^2^ per minute
Dec Abs (−3, −2)/min	n/min	Absolute decelerations from 2 to 3 m/s^2^ per minute
Dec Abs (−4, −3)/min	n/min	Absolute decelerations from 3 to 4 m/s^2^ per minute
Dec Abs (−5, −4)/min	n/min	Absolute decelerations from 4 to 5 m/s^2^ per minute
Impacts (0–3) G	n	Total impacts at intensity lower than 3 G (G = 9.8 m/s^2^)
Impacts (3–5) G	n	Total impacts from 3 to 5 G (G = 9.8 m/s^2^)
Impacts (8–100) G	n	Total impacts from 8 to 10 G (G = 9.8 m/s^2^)
Impacts (3–5)/min	n/min	Total impacts per minute from 3 to 5 G (G = 9.8 m/s^2^)
Landing (3–5)/min	n/min	Total landings per minute from 3 to 5 G (G = 9.8 m/s^2^)
Landing (5–8)/min	n/min	Total landings per minute from 5 to 8 G (G = 9.8 m/s^2^)
Landing (8–100)/min	n/min	Total landings per minute from 8 to 10 G (G = 9.8 m/s^2^)
Takeoff (3–5)/min	n/min	Total takeoffs per minute from 3 to 5 G (G = 9.8 m/s^2^)
Takeoff (5–8)/min	n/min	Total takeoffs per minute from 5 to 8 G (G = 9.8 m/s^2^)
Centr. F + MAX	N	Maximum centripetal force (left size)
Centr. F + AVG	N	Mean centripetal force (left size)
Centr. F − AVG	N	Mean centripetal force (right size)
Centr. − MIN	N	Maximum centripetal force (right size)

**Note.** m = meters; m/min = meters per minute; km/h = kilometers per hour; m/s^2^ = meters per second squared; n = count; n/min = count per minute; N = newtons.

**Table 2:** Principal component analysis in the total game and by quarters.

	Total Game					First Quarter (Q1)								Second Quarter (Q2)							Third Quarter (Q3)						Fourth Quarter (Q4)					
Eigenvalue		13.50	10.99	8.58	6.04		12.14	7.54	6.50	6.11	5.75	5.02	4.74		14.59	11.86	8.97	6.80	6.56	6.25		11.17	9.09	6.84	5.98	4.91		15.11	10.41	7.16	5.80	5.29
% Variance	21.99	35.50	46.49	55.07	61.10	21.38	33.52	41.06	47.56	53.67	59.43	64.45	69.19	18.51	33.10	44.96	53.92	60.72	67.27	73.53	31.25	42.42	51.51	58.34	64.32	69.22	24.21	39.32	49.73	56.89	62.69	67.98
PC	1	2	3	4	5	1	2	3	4	5	6	7	8	1	2	3	4	5	6	7	1	2	3	4	5	6	1	2	3	4	5	6
Expl dist	0.898					0.645										0.722							0.834									
Vel Abs (0–6) (m/min)															0.892						0.767											
Vel Abs (18–21) (m/min)									0.700							0.811							0.803							0.809		
Vel Max																																0.709
Acc/min		0.895						0.849							0.922						0.892										0.847	
Dist Acc						0.719										0.731							0.725									
MAX Acc (m/s^2^)																			0.721													
Acc Abs (0–1)/min		0.953																			0.946											
Acc Abs (1–2)/min															0.757																0.821	
Acc Abs (3–4)/min						0.838																						0.755				
Acc Abs (4–5)/min					0.794					0.841							0.894									0.820		0.742				
Acc Abs (5–6)/min													0.863															0.710				
Acc Abs (6–10)/min												0.890																				
Dec Abs (−1. 0)/min		0.960																			0.957											
Dec Abs (−2, −1)/min								0.857																								
Dec Abs (−3, −2)/min	0.775																															
Dec Abs (−4, −3)/min						0.741																										
Dec Abs (−5, −4)/min																	0.862															
Impacts (0–3) G																						0.748										
Impacts (3–5) G				0.767																												
Impacts (8–100) G																													0.746			
Impacts (3–5)/min	0.775																															
Landing (3–5)/min			0.701				0.778							0.772										0.804			0.798					
Landing (5–8)/min			0.775				0.755							0.702													0.732					
Landing (8–100)/min																									0.842				0.564			
Takeoff (3–5)/min			0.783											0.835													0.842					
Takeoff (5–8)/min			0.717																					0.817			0.735					
Centr. F + MAX				0.701							0.803									0.764									0.633			
Centr. F + AVG																				0.857												
Centr. F − AVG																		0.850														
Centr. − MIN				0.703							0.785							0.891				0.701							0.705			

**Note.** PC = Principal Component; Expl Dist = Explosive Distance; Vel Abs = Velocity Absolute; Acc = Acceleration; Dec = Deceleration; Dist Acc = Distance covered during acceleration; MAX Acc = Maximum acceleration; Centr. F = Centripetal Force; MAX = Maximum; AVG = Average; MIN = Minimum. Loading values > 0.60 are shown. Numbers in parentheses indicate intensity thresholds (e.g., 0–6 m/min, 3–4 m/s^2^). G = gravitational force units (9.8 m/s^2^).

**Table 3:** Principal component analysis between playing positions.

	Central Players				Forward Players					Guard Players						
Eigenvalue		12.78	10.65	7.06		12.86	9.45	6.78	5.67		10.89	9.25	7.214	6.80	5.39	5.00
% Variance	34.12	46.89	57.55	64.61	30.360	43.22	52.67	59.46	65.12	24.75	35.64	44.89	52.10	58.90	64.25	69.25
PC	1	2	3	4	1	2	3	4	5	1	2	3	4	5	6	7
Explosive distance					0.810											
Vel Abs (18–21) (m/min)	0.772				0.760										0.760	
Vel Abs (21–24) (m/min)															0.744	
Acc/min		0.867												0.831		
Dist Acc	0.887				0.736											
Vel Max								0.788								0.710
Acc Abs (3–4)/min	0.892				0.733					0.864						
Acc Abs (4–5)/min									0.869				0.883			
Dec Abs (−2, −1)/min		0.894					0.739							0.780		
Dec Abs (−5, −4)/min													0.792			
Impacts (0–3) G											0.761					
Landing (3–5)/min						0.741						0.738				
Landing (5–8)/min						0.831						0.701				
Landing (8–100)/min				0.839												
Takeoff (5–8)/min						0.791						0.831				
Centr. F + MAX			0.747								0.781					
Centr. F − MIN			0.762								0.774					

**Note.** PC = Principal Component; Explosive distance = Distance covered at explosive intensity; Vel Abs = Velocity Absolute; Acc = Acceleration; Dec = Deceleration; Dist Acc = Distance covered during acceleration; Vel Max = Maximum velocity; Centr. F = Centripetal Force; MAX = Maximum; MIN = Minimum. Loading values > 0.60 are shown. Numbers in parentheses indicate intensity thresholds (e.g., 18–21 m/min, 3–4 m/s^2^). G = gravitational force units (9.8 m/s^2^).

## Data Availability

The data presented in this study are available upon request from the corresponding author. The data are not publicly available due to the Organic Law 3/2018, of 5 December, on the Protection of Personal Data and Guarantee of Digital Rights of the Government of Spain, which requires that this information must be in custody.

## Abbreviations

The following abbreviations are used in this manuscript:

ANGT	Adidas Next-Generation Tournament
BMI	Body Mass Index
EPTS	Electronic Performance and Tracking Systems
G	Gravitational Force (9.8 m/s^2^)
GPS	Global Positioning System
Hz	Hertz
IMU	Inertial Measurement Unit
KMO	Kaiser–Meyer–Olkin
LPS	Local Positioning System
MDS	Most Demanding Scenarios
MEMS	Microelectromechanical Sensors
PCA	Principal Component Analysis
PC	Principal Component
Q1	First Quarter
Q2	Second Quarter
Q3	Third Quarter
Q4	Fourth Quarter
sRPE	Session Rating of Perceived Exertion
TDOA	Time Difference of Arrival
UWB	Ultra-Wide Band
